# Supplementary material for: Examining Conjoint Behavioral Consultation to Support 2e-Autism Spectrum Disorder and Gifted Students in Preschool with Academic and Behavior Concerns
Source: Behav Sci (Basel). 2023 Aug 11;13(8):674. doi: 10.3390/bs13080674 (PMC10451274; doi:10.3390/bs13080674)
Supplement: Supplementary file 1 [file behavsci-13-00674-s001.zip › behavsci-2475036-supplementary.pdf]

Supplementary Materials

1. Word Problem Samples

Change type word problem: Bob had a lot of meals ready for his long walk that he had planned. He loaded all the meals on his donkey and as he trekked over the highlands, he managed to eat 42 separate meals. When he reached the end of his trek, he still had 15 dinners remaining. How many meals did he pack for his trip into the wilderness?

Group type word problem: On his farm, Farmer has a total of 88 animals. Horses and goats are his only livestock animals. On the farm, there are a total of 49 horses. On the farm, how many goats are there in total?

Compare *type word problem*: One of the two dolphin brothers is 90 cm. His younger brother is 10 cm shorter. How tall is the little dolphin?

Art: Students find the desired animal in the picture and then create their own pictures in the artistic style they want. Search and Find Picture Puzzles (Ormanda/In the forest, ISBN 978-605-312-465-8, Hayvanat Bahçesinde/ In the Zoo, ISBN 978-605-464-1).

2. Science

The teaching content for science was prepared in two stages. The examples in the first-level (2.1.) interests of students with ASD constitute the examples. 2.2. section contains second level examples. Examples at the second level include children's access to deeper information in their areas of interest and topics that they are curious about and want to learn more about at the first level. For example, "How do mammalian (mother) whales give milk to their babies underwater?"

2.1. *Habitats:* Many creatures also live on land. Areas such as forests, mountains, meadows, seas and lakes are the habitats of some living things on land. Creatures such as cats, dogs, goat, horse, donkey, bears and cows live on land.

There are also creatures that live both on land and in water. Animals such as frogs, hippos, crocodiles, turtles, and beavers are also animals that can live both on land and in water.

Many creatures also live in water.

Animals living in the specified ecology:

Characteristics of animals living in the determined ecology:

Fish, Crab, Seal, Octopus, Shark, Whale, Dolphin, Pelican

Fish Characteristics

It breathes with its gills.

It cleans the dirty blood with its gills.

It is usually scaly.

Saltwater fish drink water. Freshwater fish do not drink water.

Fish species are quite diverse. There is a wide variety of fish that can emit light and fly for a short time.

Crab Characteristics

It has double clamps.

He walks sideways.

It is both herbivorous and carnivorous.

His body is extremely robust.

The abdomen is small.

Seal Features

They have strong nails.

Hears very well in water and air.

Food sources are diverse. It feeds mostly on fish that live at depth.

Octopus Features
It has big eyes.
It has 8 arms.
He is an expert at hiding.
It is extremely clever.
Its color can adapt to its surroundings.
He can run fast.
Its body is very soft.

Shark Features
Its skin is usually pale gray and very hard.
He is muscular.
It has an upturned tail.
It has to swim constantly in order not to sink.
He has incredibly good vision.

Whale Features
He is warm.
She nurses her baby with milk.
Some have a small dorsal fin.
It plans extremely cleverly to find its prey.

2.2. *Explore*

J.F. Turner. Dietary Habits of The Blue Whale. Retrieved on: 16 July 2020. Retrieved from: Animal Wised | Archive Link

B. Garrod. How Do Whales Breastfeed Underwater? Retrieved on: 16 July 2020. Retrieved from: Discover Wildlife

Dolphin Features
His skin is like rubber.
It has a mixture of black, white and gray color.
He is curious and playful.
He sleeps by resting one side of his brain.
It can jump up to 6 meters out of the water.

Pelican Features
It has broad wings.
His neck is quite long.
It has a huge beak.
Its legs are very small compared to its beak.
It uses its beak for fishing.
Small group work: Preliminary work to carry out ecological site selection
